# Statistics of Crime, and the Moral and Mental Condition of Prisoners

**Published:** 1852-10-01

**Authors:** 


### Art. III.—STATISTICS OF CRIME, AND THE MORAL AND MENTAL CONDITION OF PRISONERS.

We have before us the seventeenth report of the *Inspector of the Prisons of Great Britain,* and the last parliamentary report relating to the *Discipline and Management of Pentonville, Parkhurst, Millbank, Portland, and Dartmouth Prisons, including official returns of the condition of the Hulks for the year* 1851. This document contains much valuable and interesting matter, illustrative of the state of our criminal population *in duresse.* We purpose, however, confining our attention to the moral state and mental health of the prisoners; and our extracts from the documents under review will relate exclusively to these points. Under the head of "Religious and moral instruction," the chaplain of the Pentonville prison makes a satisfactory report. When speaking of the educational condition of the prisoners, the chaplain observes, that they had at least had as good educational *opportunities* as the classes in society to which they belonged, but to which in the matter of honesty and good conduct they were so disgracefully the exceptions; and therefore that the mere want of secular education was not the cause, or even a chief one, of their crime, which being removed, they would naturally return to society reformed and useful characters. Some proved to have been highly educated, and a very large number sufficiently so for any situation of life which they were ever likely to occupy.

These facts rather militate against the generally entertained opinion, that the criminals of this country belong to the uneducated classes.

In regard to the mere improvement of the convict, he says, that during the past year there has been less *profession* of a religious character. But the chaplain thinks the results have not been worse on this account. Experience has taught him to be more cautious in receiving as undoubted, professions of a change of heart in men shut out so completely from the great temptations of life. The tenour of the addresses would therefore insensibly, perhaps, partake more of this character, and the prisoner would be led thereby rather to defer his profession of such a change until placed in more natural circumstances.

There is much good sense in the following remarks :—

"I think nothing easier for an affectionate, zealous Christian minister to accomplish, than to move the feelings of prisoners in separation, and to gain control almost over their very wills; but it would be a great mistake to conclude that there was a real change of the principles or character of the man whenever this effect is produced. That, under God, this influence may tend to salvation in some cases, and may civilize and bring men into order to a very great extent, I thoroughly believe; but, as regards real change of heart, I must say, looking closely into such accounts as have been made public of changes wrought in large proportions of prisoners, they require to be received with considerable allowance for partiality and a sanguine temperament in the writers. Real conversions to God are not more frequent in our prisons than in our refuges and penitentiaries; and compared with the general mass of people, under similar advantages of Christian faithfulness, zeal, and love, I believe them to be more rare. Nevertheless, few prison chaplains of this character are without the encouragement of knowing that their labour is not vain in the Lord; whilst it must not be lost sight of, that Christianity, if it accomplishes nothing more, civilizes man, subdues ferocity, procures respect for laws, improves the mind, enlarges and elevates its conceptions, and gives vigour and judgment to the conscience. This aspect of religion is constantly presented to our view. During the past year we have had reason to be encouraged in both these respects."

The medical officer's report of the state of the Pentonville prisoners is an important document. We designedly confine our attention to the *mental* health of these who have, during the year 1851, been confined in this establishment.

Two cases of insanity, requiring removal to Bethlem, have occurred during the year. Their history is subjoined.

James Satchwell, 3259, was received from the Hulks, where he had obtained a very bad character on account of repeated acts of insubordination and violence.

On several occasions during his imprisonment in Pentonville, he exhibited similar misconduct, and committed breaches of discipline so flagrant as to lead to doubts being entertained as to his actual sanity. He had naturally but low intellectual development, and his cranial capacity was found, by actual measurement, to be far below the average. He could neither read nor write, and was incapable even of learning a trade. In disposition he was cunning, suspicious, and deceptive.

After much close observation, it was acertained that the prisoner was the subject of insane delusions, and he was therefore removed to a lunatic asylum. It was impossible, however, for a time, in the absence of the evidence of positive delusion, to state that he was irresponsible for his actions, or to say that his violent conduct was not alone attributable to a wicked and ungovernable temper.

The history of the other case is as follows :—

B. Woller, 3,688, aged 24, of bad moral character, having been three times previously convicted and imprisoned, and several times also examined on charges of felony, and discharged on account of defective evidence; was noticed on admission as somewhat pallid; he had also some imperfectness of vision, accompanied by a peculiar and constant oscillation of the eyeballs. He had been but three weeks in the prison, when he was suddenly seized with mania, and was shortly afterwards removed to Bethlem.

The morbid conditions noticed on admission were regarded at the time as of functional origin only; but, considered in connexion with the subsequent attack of insanity, it is presumed both may be referred to cerebral disease which existed previous to his imprisonment.

The number of removals to Bethlem, as compared with preceding years, is found to be :—

·27 per cent. on the prison population of the first seven years.
·32 per cent. on the prison population of 1850.
·16 per cent. on the prison population of 1851.

Three cases of delusion have also occurred :—

1. W. Ross suffered from depression, and was the subject of aural delusions. These symptoms disappeared under treatment, and he was subsequently removed as unfit for further separate confinement.

2. H. Litchfield laboured under the delusion that he was suspected of having committed an unnatural offence, and that this was made a subject of conversation by the warders. The delusion in this case had ceased to affect the prisoner before he was removed from the prison.

3. H. S. M'Laughlin, 3,617, has been affected with insane delusions, which, under association, have vanished at times, and again re-appeared. This case also recovered under treatment in the prison.

Besides the cases of insanity and delusion already noticed, there were observed other cases, in which mental depression, irritability, natural feebleness of intellect, and other conditions not amounting to actual disease, existed, and rendered relaxation, or suspension of the discipline, necessary in the first place, and removal from the prison advisable at a subsequent period. They amounted in all to 22.

The report from which the preceding facts have been gleaned, is made by Mr. C. Lawrence Bradley.

*Parkhurst* prison next claims our attention. It appears from the chaplain's report, that he had under his own observation, in the year 1851, 280 juvenile prisoners. The subjoined facts constitute the result of his examination of 154 general ward boys. It appears that only 62 of them had both parents alive at the time of their conviction; 30 had neither father nor mother, and the remainder had but one of the parents living: so that 92 out of the 154 were orphans,—a fact calculated to excite pity. If industrial orphan schools were established, which, by all accounts, might be made to support themselves, a vast amount of crime would be prevented, and great expense saved to the country.

104 had attended day schools, for periods of from six months to seven years. Few of them were at school less than one year, so that a want of education,—or rather, to speak accurately, a want of the means of instruction,—was *not*, in the case of these boys, the cause of crime; and the chaplain begs to be allowed to express his conviction, that it is not that kind of education which, in the present day, is so much sought to be given to the children of the poor, that will prevent crime, and make its recipients respect the rights of others. His experience here and elsewhere shows that what we need is, such a course of *industrial* training, combined with religious and secular instruction, energetically imparted, as shall fit its recipients for the station they are likely to occupy, and the duties they will probably have to perform. His observation does not show him that what is now called education accomplishes this. Four have only attended ragged schools. As large a proportion as 113 have been Sunday scholars, for periods ranging from three months to six years. Only 7 resorted to thievery from poverty and want. It appears that 3 only out of the 154 were led into crime, or encouraged in it by the father, and not one, in either respect, by the mother.

It will be seen, that, from the above particulars, it may be inferred that the prisoners last year received did not come from the lowest classes. The question then occurs, What was the cause that led these boys into crime? The *immediate* cause was bad company. As many as 137 attribute their fall into crime to this cause; and the truth of this is manifest from their general history. It coincides, too, with another point, that as many as 135 came from large towns and populous places; just those parts where the greatest facilities exist for getting into this sort of company. The rest came from small towns. We seldom get a prisoner from a really rural and scattered population.

But the *remote* cause was a want of proper parental control: only 62 had both parents alive to control them; all the rest were deprived either of both parents, or of one. But those parents that are alive exercised little or no authority over these their children, but allowed them to be out late at night, with those ill-behaved and loose characters that congregate in the streets; and there it is that they are picked up by those who are already thieves, and who are always on the look-out to recruit the ranks which justice has thinned. They are then treated by these prowlers to the low theatres, and to feasting at a public-house, until they are entangled in the net of the destroyer. Thus it is that more than two-thirds of juvenile crime are propagated; indeed, so prolific of crime is this unobserved cause, and such misery and expenditure does it entail, that it is worthy of universal notice.

In connexion with the last particular, no more than 5 out of the 154 ever went to an evening school; thus we arrive at the same point from a different direction, for if they had been at an evening school they would not have been open to the pernicious influence above referred to.

The following table will show accurately how many times each boy has been in prison:—

| | | | | | |
|---|---|---|---|---|---|
| 14 boys | . | not once in prison. | | | |
| 27 „ | . | 1 | equal to | 27 | imprisonments. |
| 23 „ | . | 2 | „ | 46 | „ |
| 24 „ | . | 3 | „ | 72 | „ |
| 15 „ | . | 4 | „ | 60 | „ |
| 12 „ | . | 5 | „ | 60 | „ |
| 10 „ | . | 6 | „ | 60 | „ |
| 7 „ | . | 7 | „ | 49 | „ |
| 2 „ | . | 8 | „ | 16 | „ |
| 1 „ | . | 9 | „ | 9 | „ |
| 3 „ | . | 10 | „ | 30 | „ |
| 3 „ | . | 12 | „ | 36 | „ |
| 1 „ | . | 14 | „ | 14 | „ |
| 1 „ | . | 15 | „ | 15 | „ |
| 3 „ | . | 18 | „ | 54 | „ |
| 8 „ | . | not known | | 0 | „ |
| | | Total | . | 548 | imprisonments. |

146 of these boys were, before their sentence to transportation, imprisoned no less than 548 times, while the mischief they did to the morals and property of others is incalculable. Does not this suggest how very much more economical it would be to prevent juvenile crime, by an industrial and religious training, than after allowing it to be manufactured and perpetrated, to reform it?

The improvement in the moral conduct of the prisoners during the past year was greater than in any previous year; and this not only in one, but in all departments of the prison. The governor in his report gives the statistics of behaviour in the wards; the following refer solely to the conduct of prisoners during the time they were in school.

| | |
|---|---|
| Total number of boys under instruction during the course of the year | 632 |
| Number of boys entered into the school misconduct-book for petty offences | 70 |
| Number of boys reported to the Governor for continued petty misconduct, or more serious offences | 71 |
| Number of boys who have not been complained of at all in school throughout the year | 562 |
| Number of boys who have not been reported to the Governor throughout the year | 561 |

Number per cent. :—

| | |
|---|---|
| 1. Of boys complained of for petty offences | 11 |
| 2. Of boys not complained of at all | 89 |
| 3. Of boys reported to the Governor | 11 |
| 4. Of boys not reported to the Governor | 89 |

Of the 70 boys entered in the school misconduct-book for petty offences, there were complained of, once, 38; twice, 10; three times, 10; four times, 6; six times, 1; eight times, 1.

Of the 71 boys reported to the governor, there were reported, one, 53; twice, 10; three times, 4; six times, 1.

We have, under the above heads, a total number of 250 offences, but 201 of these offences were committed by the boys while they were in the probationary ward—that is, during the first four months of their residence here; a pleasing indication of the efficiency of the means used for their improvement.

On the 1st of November last, a new general routine came into operation, by which it was arranged that boys who had been here a certain time should altogether cease attending the day school, and be always industriously occupied, as it was conceived that this would better prepare them for labour in the colonies. The *principle* of this alteration is good, and in the right direction; and it seems capable of producing useful results if properly carried out.

In May of last year, another change took place in the same beneficial direction, by which it was arranged that the prisoners should attend school one morning of one day of the week for four hours, and one afternoon of another day of the week for five hours; that thus, even on school days, the prisoners might have either a long morning or a long afternoon for labour.

Stimulants to industry and good conduct were at the same time added. All who have been here a sufficient length of time, and whose correct behaviour has merited a good-conduct badge, are allowed a slice of pudding on the Sunday, if they have worked diligently and behaved well during the preceding week. They have also a small amount of their earnings credited to their account, to be received by them when they get to the colonies. But the reward which is most prized is the liberty of writing to their friends at stated seasons.

"In allowing these rewards, it was considered," says the chaplain, "that to expect prisoners 'to do well for its own sake,' was to suppose them to have arrived at such a state of virtue which, if it had been possessed, would have effectually prevented them from committing the crimes for which they are punished. This motive is all-important as the ultimate one to be aimed at, but it is inapplicable to those who are utterly destitute of that moral sense by which alone it can be appreciated; and I would with reverence suggest, whether the Great Ruler of the world does not hold out present secular advantages, as well as future spiritual rewards, in His infinitely able system of reforming mankind and preparing them for another sphere of happy existence. In my judgment, therefore, gentlemen, you acted wisely in recommending these rewards, and the present state of the prison justifies the measure."

"The numbers of the prisoners that act well from Christian motives give a joy to the chaplain which, as it is not generally appreciated, he will do no more than allude to, though it is one great stimulus to his exertions in the midst of frequent and extreme depression of spirits, necessarily arising from the peculiar and monotonous nature of his duties."

The following extract from the chaplain's report will convey to our readers an accurate idea of the kind of religious instruction provided for those confined in the *Millbank* Prison :—

"During different periods, amounting to six months in the course of the year, the number of schoolmasters employed was seven, at other times eight, the present number.

"The whole of the male prisoners not in the infirmary, or hindered by casual sickness, with the following exceptions, those permitted to be absent on account of dissent from the Established Church, employed as artisans, kept back for the morning school, engaged in the bakery, and some of those employed in the kitchens, have regularly attended the daily chapel service.

"On Sundays the prisoners in association have attended two full services; those in separation, the morning and afternoon alternately; those employed in the bakery have had the benefit of both services; arrangements have been lately made for prisoners in association receiving further religious instruction in the wards, from the schoolmasters, on each alternate Sunday evening.

"The assistant chaplain has been in the regular practice of visiting the infirmary wards every day, *reading prayers in all,* and delivering a *lecture* to the prisoners in *each successively.*

"On Sundays suitable ministrations have been afforded in the infirmary, by the religious instructor reading both prayer and lecture in its several wards.

"The religious instructor has on week-days been constantly engaged in reading the Holy Scriptures with the prisoners from cell to cell, and giving catechetical instruction to the juvenile prisoners individually.

"The Holy Communion has been administered to the male prisoners four times in the year, the communicants having been previously visited by the chaplains, with a view to their suitable preparation.

"The behaviour of the prisoners, as falling under the chaplain's observation, has been orderly; and the schoolmasters speak satisfactorily of the attention generally evinced. Of *moral improvement*, however, as regards the *many*, embracing change of principle, and *real amendment of character*, he feels considerable diffidence; bearing in mind the circumstances of the prison—the period of separate confinement, rarely exceeding six months, being somewhat brief to be *permanently effective* for reformatory purposes—the danger of any good impressions made during that period (the seedtime of reformation) being effaced when prisoners are transferred to the *large rooms and general ward*, where the opportunity is withdrawn from those under incipient convictions of being ever *left alone with their conscience*, and the spiritual exercises of the more advanced in religion, both meditation and prayer are subject to disturbance."

The number of insane cases during the year 1851 is, we regret to find, considerably above the average; whilst referring to this fact, the medical officer observes that it is his opinion that there are no new causes tending to injure the minds of the prisoners in operation at Millbank. Of the eight prisoners removed to Bethlehem Hospital, five, Dr. Baly says, were decidedly insane when admitted at Millbank. Two others were noticed at the time of their reception to be of very low intellect, one of them also sullen, and in a state already verging on insanity.

We append, without abridgment, Dr. Baly's important statement of facts. It will be perused with interest by thousands whose attention has been zealously directed to the consideration of the influence of prison discipline and confinement on the health of the mind:—

"Thomas Whittaker, who had been convicted of an unnatural crime, appeared from the time of his reception weak in intellect, was idle, inattentive to all rules for order and cleanliness, noisy, frequently disturbing the ward by singing and whistling, and, when admonished, most violent and abusive in his language. Unequivocal symptoms of insanity showed themselves on the 23rd October, after he had been eight months in prison.

"The only patient removed to Bethlehem Hospital who was received in a sound state of mind, was Ann Moran. This prisoner was received from York, on the 28th March, 1849. Her insanity declared itself in the form of violent mania, on the 28th March, 1851; but for some weeks previously, she had been strange in conduct, pilfering various articles of clothing, &c.; concealing them in her bed, telling falsehoods without any apparent motive, &c.

"Five patients who suffered from different forms and degrees of disordered intellect were not removed to a lunatic asylum. One of these,

"William Lamb, received from Aberdeen, was in a state of complete dementia at the time of his reception; another,

"Daniel Coghlin, received from Manchester, on the 13th February, 1851, was a man of naturally weak intellect, and timid disposition. From the middle of September, 1851, he laboured under delusions, characterized chiefly by fear of injury to himself. He has recovered.

"The three following were apparently of sound intellect at the time of their reception into the prison; although one of them had been insane previously :—

| | | | |
|---|---|---|---|
| Cain Squires . . . . | received from | | Wakefield. |
| Henry Smith . . . . | „ | „ | Warwick. |
| Henry Scull . . . . | „ | „ | Bristol. |

"Cain Squires, who was received on the 15th February, 1851, began to manifest some disorder of intellect in May. His only delusion related to his trial and sentence. Under the influence of this delusion, he has at times been restless and excited. But usually he has been quite tractable, and during long intervals has apparently recovered his soundness of intellect.

"Henry Smith was received into the prison on the 13th February, 1851. He was a youth of considerable intelligence. His delusions, which for the most part have religious questions for their subject, unfortunately still persist; although he is in good bodily health, and retains perfect clearness of intellect on general matters.

"Henry Scull was sent to Millbank prison from Bristol on the 28th of January, 1851. He began to talk incoherently at the commencement of August, soon afterwards became violent and destructive, and then lapsed into a state of dementia. Within the last two months his state of mind has so much improved as to give strong grounds for hope that he will speedily recover. His removal to Bethlehem Hospital, an order for which had been obtained, has, consequently been postponed. His parents, who visited him during his insanity, stated, that he had twice before been insane, with the same symptoms, and had even required restraint.

"With respect, then, to the origin of the cases, the facts are, that of 13 insane patients, 6 were at the time of their reception insane, 3 of weak mind, and 4, including the female patient, of sound mind. Extending the same inquiry to all the cases of insanity that have occurred in the prison amongst the male prisoners during the last eight years, we obtain the following results :—

| | Average daily Number of Male Prisoners. | Total Number of Insane. | Insane when received. | Of weak Mind when received. | Of sound Mind when received. |
|---|---|---|---|---|---|
| Year 1844 . . . . | 699 | 3 | 3 | — | — |
| „ 1845 . . . . | 828 | 4 | 1 | 1 | 2 |
| „ 1846 . . . . | 845 | 8 | 3 | — | 5 |
| „ 1847 . . . . | 983 | 7 | 4 | 2 | 1 |
| „ 1848 . . . . | 1223 | 11 | 7 | 1 | 3 |
| „ 1849 . . . . | 896 | 15 | 10 | 1 | 4 |
| „ 1850 . . . . | 1001 | 6 | 1 | 2 | 3 |
| „ 1851 . . . . | 918 | 11 | 6 | 2 | 3 |
| Aggregate numbers for the eight years . . | 7393 | 65 | 35 | 9 | 21 |
| Annual proportion of insane of each class, per 1000 prisoners . . | . . | 8·75 | 4·73 | 1·21 | 2·84 |

"During the period specified, 65 male prisoners have come under treatment for insanity in the prison; but of this number 35 were insane when they were received, and 9 were of weak mind, or, in some instances, in a state verging on insanity. Only 21 were in quite a sound state of mind when they came into the prison; and if we compare these numbers with the male population of the prison during the same period, we find that the annual proportion of cases of insanity in patients previously of sound mind was somewhat less than 3 (2·84) per 1,000 prisoners; and, including the cases of patients previously of weak mind, more that 4 (4·05) per 1,000 prisoners. This at first sight appears a higher rate of cases of insanity than would be expected in the Millbank prison, where, for several years past, only about two-thirds of the male convicts have been confined in separate cells, and where the average terms of imprisonment in the several years have ranged between 2½ months and 6½ months; but before the numbers above given are left for comparison with the statistics of other prisons, it must be explained that they include the slighter forms of disordered mind, consisting in "delusions," and that many of the patients recovered in the prison, several after very short attacks. From the subjoined table it will be seen, that, of the 30 prisoners attacked with insanity during their imprisonment (those previously of weak mind or doubtful sanity being included), 14 recovered without removal to a lunatic asylum, or other place of confinement. The remaining 16, who did not thus speedily recover, or who died, give an annual ratio of only 2 (2·16) per 1,000, when compared with the number of male convicts in the prison during the eight years. And this ratio, though double that of the cases of insanity of similar severity which occur annually amongst the general population out of prison, is not greater than might perhaps be anticipated from the nature of the various influences acting on the minds and bodies of convicted criminals. That the length of the imprisonment, up to a certain limit, affects the result considerably, is quite certain, and is rendered sufficiently apparent by the following facts:—

| | | Recovered in the Prison. | Removed to Bethlehem or other Prisons or Asylums. | Died. | Remarks. |
|---|---|---|---|---|---|
| Insane Prisoners of weak mind when received . . | 9 | 1 | 6 | 2 | One committed suicide, and one died of dementia. |
| Insane Prisoners of sound mind when received . . | 21 | 13 | 7 | 1 | Died of bodily disease unconnected with his insanity. |
| Both Classes . . . | 30 | 14 | 13 | 3 | |

"During the former four years of the period above referred to, the average duration of the imprisonment of the male convicts was only three months and seven days; and the number of cases of insanity amongst them was 11, or 3·28 per 1,000 prisoners annually. During the latter four years (1848 to 1851 inclusive) the average duration of their imprisonment was five months and six days; and the number of cases of insanity was 19, giving an annual ratio of 4·70 per 1,000 prisoners. Omitting the cases of those who recovered in the prison, the number of cases in the former period becomes five, and the annual ratio 1·49 per 1,000; the number in the latter period 11, or 2·72 per 1,000 annually.

"The same result, namely, the increasing risk to insanity that attends the protraction of imprisonment, at all events through the first 12 months, is shown more precisely in the subjoined table. It will be seen that of 30* male prisoners who became insane in Millbank prison in the course of the last eight years, only 9 were attacked during the first three months of their imprisonment, 9 in the course of the second three months, 8 in the course of the third three months, and 4 at later periods; while about 16,000 prisoners passed through a single three months' imprisonment, only about 8,400 through a second three months' imprisonment, about 4,200 through a third three months, and about 1,200 through a fourth three months: so that the ratio of cases of insanity has been nearly twice as high in the second three months of imprisonment as in the first three months, and in the third three months more than three times as high as in the first.

* In three cases (L. D., J. J., and M. M'G.) the men were so exceedingly imbecile or melancholic at the time of their reception, and passed so gradually into a state of complete insanity, that the period of the commencement of their attacks cannot be exactly determined. Two of these cases are referred to the first three months, and one to the second three months of imprisonment.

| Periods of Imprisonment. | Approximative Number of Prisoners who passed through each Period. | Number of Cases of Insanity occurring in each Period. | Annual ratio per 1,000 of Cases of Insanity for each Period. |
|---|---|---|---|
| First Three Months . . . . . . | 16,000 | 9 | 2·25 |
| Second Three Months . . . . . | 8,400 | 9 | 4·28 |
| Third Three Months . . . . . | 4,200 | 8 | 7·61 |
| Fourth Three Months, or later . . | 1,200 | 4 | — |

"With respect to the particular influences which give rise to mental disorder with this increasing frequency during the first nine or twelve months passed in prison, it may, I think, be affirmed that the more powerful ones are included under the following heads:—

"1. The various feelings of remorse, shame, and despondency, which act most strongly on educated and sensitive minds, and at an early period.

"2. The withdrawal of the accustomed external sources of excitement inducing a state of inertia, or torpor of mind, which leaves any tendencies to mental disease more free to develop themselves. This cause affects more frequently men of low intellect and deficient education, and, in others, produces its evil results at a later period.

"3. Various morbid influences acting on the mind through the body; including many disturbances of the general health, due chiefly to comparative deficiency of exercise and fresh air, and the exhaustion of nervous power induced by the 'solitary vice;' this last cause operating especially on young persons, and generally after some months' imprisonment.

"All these influences, but especially the latter two, will, it is obvious, have greater sway in proportion as the imprisonment involves more of actual seclusion. It is, therefore, not surprising that at Millbank prison during the last eight years the cases of insanity have been much less numerous among the prisoners 'associated' in large rooms, than among those confined in 'separate' cells. The number of prisoners in separate cells has, on the average, been 624; the number in association 298. The cases of insanity among prisoners of the separate class have numbered 24; the cases among those of the associated class have been 6. The annual ratio of cases of insanity has, consequently, been only 2·52 per 1,000 among the latter, and 4·78 per 1,000 among the former.

"An important fact remains to be noticed, namely, the much larger proportion of men originally of weak or dull intellect among the prisoners who became insane while in separate confinement. Of the 6 prisoners attacked with insanity while in 'association,' only 1 was weak-minded at the time of his reception; while of the 24 men who became insane in separate cells, 8 were imbecile, or of a very low grade of intellect. If, now, these men of originally weak mind be excluded from the calculation, the preponderance of cases of insanity among the prisoners in 'separation' is, of course, much diminished; for there remain only 16 cases of insanity amongst prisoners of the separate class, or 3·19 per 1,000 annually; and 5 cases among those of the associated class, or 2·10 per 1,000 annually. From the calculation thus made, the inference at first sight appears to be deducible, that men of sound mind do not suffer in any very considerable degree more from separate confinement than from imprisonment in 'association,' but in more or less strict silence. But such a conclusion would be erroneous: for the average term of imprisonment of the prisoners of the 'associated' class at Millbank prison has been far longer than that of the prisoners of the 'separate' class; the 700 prisoners of the juvenile class, who in the years 1844 to 1848 underwent on the average 12 months imprisonment, and nearly all the prisoners who on account of physical disabilities or other causes have been detained for long periods in the prison, except the 'incorrigibles,' having been in 'association.' So that if the influences tending to disturb the intellect acted equally under the two modes of imprisonment, the proportion of insane ought to be much greater among the prisoners in 'association;' while, notwithstanding their longer terms of imprisonment, it has been one-third less.

"The above-mentioned facts do, however, show incontestably the great danger that attends the confinement of prisoners of weak minds in separate cells. It might, I think, almost be affirmed that men of any considerable degree of imbecility, or great dulness of intellect, will with certainty be rendered actually insane or idiotic by a few months separate confinement; and the multiplication of cases of insanity at Millbank prison, where so many men of impaired or deficient mind are received, has been prevented only by the precaution of placing in association all such prisoners as soon as their infirmity of mind became known to the medical officer.

"The man who committed suicide on the 25th September, 1851, after having been eight months in the prison, had been convicted and sentenced to transportation for an unnatural offence. He had manifested no symptoms of insanity, nor any great despondency, and the only probable motive for the act was the fear of immediate transportation, since he had been on the previous day inspected, together with other prisoners, who were about to be removed from the prison."

The following carefully written analysis of "The Seventeenth Report of the Inspectors of Prisons of Great Britain," we copy from the pages of a contemporary.* It appears to embody all the facts of any interest contained in the parliamentary document:—

"The criminal tables afford pleasing evidence that the decrease of crime, as compared with the amount ten years ago, continues to be

* *The Athenæum.*

maintained. For, although the slight increase of 4·2 per cent. marks the returns of 1851 as compared with those of 1850, the increase of population may be most fairly adduced as a satisfactory cause for this increase. The commitments during the last ten years stand thus :— 1842, 31,309 ; 1843, 29,591 ; 1844, 26,542 ; 1845, 24,303 ; 1846, 25,107 ; 1847, 28,833; 1848, 30,349 ; 1849, 27,816 ; 1850, 26,813 ; 1851, 27,960 : total, 278,623.

"The increase of 4·2 per cent. during the past year has not been confined to any particular localities. It extends generally over England and Wales, including all the chief agricultural and the largest manufacturing and commercial counties. In 1841 the commitments were in the proportion of one in every 573 of the population, while, according to the last Census Returns, the proportion in 1851 is reduced to one in 641. Between these two periods the population increased 12·6 per cent., while the commitments remained as nearly as possible stationary, their increase amounting only to a fraction per cent. But the relative progress of population and crime has been very different in different parts of England. In the large manufacturing districts where the working-classes during the past year have been steadily employed, the proportion of commitments to the population has signally decreased. Thus, in Yorkshire and Lancashire, the population during the last ten years has increased 18·2 per cent., while the commitments have simultaneously decreased 4·3 per cent. In Cheshire, Derbyshire, Nottinghamshire, and Leicestershire, where, mixed with a considerable agricultural population, the chief silk, lace, and other textile fabrics are produced, the proportion of the commitments decreased from 1 in 579, to 1 in 633, the population having increased 7 per cent. while the commitments decreased 2 per cent. In Staffordshire, Warwickshire, and Worcestershire, the seat of the chief manufactures in hardware, pottery, and glass, the commitments decreased from 1 in 435 of the population to 1 in 552, the population having increased 20·4 per cent., and the commitments decreased 5 per cent.

"In the more purely agricultural counties the progress is slower, and the results less favourable. In the eastern district, comprising Essex, Norfolk, Suffolk, and Lincoln, the proportion of the commitments to the population has increased from 1 in 669 to 1 in 604 ; the increase of the population being 6·8 per cent., and of the commitments 18·4 per cent. Of the seven chief midland agricultural counties, Cambridge, Northampton, Bedford, Hertford, Oxford, Bucks, and Berks, the proportion of commitments has decreased from 1 in 572 to 1 in 620; the increase of the population being 10·3 per cent., and of the commitments 1·8 per cent. only. In the counties in the south and southwest, Hants, Wilts, Dorset, and Somerset, the results prove more favourable than in any of the other agricultural districts. The proportion of the commitments to the population has decreased from 1 in 508 to 1 in 651 : the population having increased 12·5 per cent., and the commitments decreased 12·1 per cent.

"On a comparison of the offences upon which the increase of the commitments last year has arisen, it appears that the increase has extended to each of the classes of crime, with the exception of the sixth class, comprising miscellaneous offences. In the first class, *Offences against the person,* the commitments for murder, attempts to murder, wounding, &c., remain stationary; unnatural offences, however, show an increase, as do those under the head of lesser offences of assault. In the second class, *Offences against property committed with violence,* the commitments have been without change, except the marked increase of robbery, the tendency of the whole class, on a more extended comparison, being to an increase. In the third class, *Offences against property committed without violence,* which contains the great bulk of the commitments, there is an increase of three per cent., arising chiefly in the commitments for larceny from the person and for frauds. The fourth class, *Malicious offences against property,* although comprising a very small comparative proportion of the commitments, yet exhibits a marked increase, particularly under the heads of incendiarism and obstructing railway carriages. In the fifth class, *Forging, and offences against the currency,* there is a considerable increase, particularly under the head of uttering counterfeit coin, which offence has increased thirty-six per cent. on a comparison of the totals of the last two five years.

"The foregoing analysis refers to the total number of commitments during the past year; the following table shows the result of the judicial proceeding. We place the return of 1850 in juxtaposition for more ready comparison:

| | 1850. | 1851. |
|---|---|---|
| Not prosecuted and admitted evidence | 141 | 131 |
| No bills found against | 1,458 | 1,484 |
| Not guilty on trial | 4,639 | 4,744 |
| Acquitted and discharged | 6,238 | 6,359 |
| Acquitted on the ground of insanity | 26 | 13 |
| Found insane | 12 | 9 |
| Sentenced to death | 49 | 70 |
| " transportation | 2,578 | 2,836 |
| " imprisonment | 17,602 | 18,418 |
| " whipping, fine, &c. | 307 | 248 |
| Pardoned without sentence | 1 | 7 |
| Total number convicted | 20,537 | 21,579 |

The effect of the act of Parliament passed in 1849 to repeal the punishment of transportation on a first conviction for simple larceny, is more fully exemplified by the returns of last year. The capital sentences in 1851 are above the yearly average since 1841, when the last alteration of the law abolishing capital punishments took place. This increase arises chiefly on the offences of burglary and robbery, attended with personal violence or injuries. Of the 70 persons capitally convicted last year, the sentence was recorded against 53, sentence of death upon 17; and of these 17, 10 were executed, 2 of them being females. The proportion of crime among females has shown a slight tendency to increase in the last three years. The proportions are as follows:—1848, 23·4 females to 100 males; 1849, 24·1; 1850, 24·4; and 1851, 24·8.

In the offences against the person, the proportion of females last year was 13·4 to 100 males. In murder the large and increasing proportion of females, arising from the many cases of poisoning, has been much remarked. The number last year was 41 females to 33 males.

"It would be extremely interesting to compare the amount of crime with the extent of education in each county, and to be enabled to mark the extinction of the former by the growth of the latter. A remarkable instance has lately shown that crime is rife where education is neglected. In the borough of Stockport, possessing a population of 85,000, which has just made itself conspicuous for its atrocities, the reports of the School-inspector state that only 350 children were at school in the whole borough.

"We do not mean to say that education would blot out crime, but there can be no doubt of its beneficial nature; and we have hopes that our Legislature is beginning to discover that education is less expensive, and more honourable to a nation, than huge machinery in the shape of prisons, transport ships, and penal colonies for the punishment of crime."

